#  Altered plasma marker of oxidative DNA damage and total antioxidant capacity in patients with Alzheimer's disease

**Published:** 2016

**Authors:** Ameneh Moslemnezhad, Soleiman Mahjoub, Mehdi Moghadasi

**Affiliations:** 1 Student Research Committee, Babol University of Medical Sciences, Babol, Iran.; 2 Cellular and Molecular Biology Research Center, Health Research Institute, Babol University of Medical Sciences, Babol, Iran.; 3 Based Health Products Research Center, Babol University of Medical Sciences. Babol, Iran.; 4 Mehr-Avaran-Shomal Nursing Home, Sari, Iran.

**Keywords:** Alzheimer's disease, Oxidative stress, DNA damage, 8-OHdG, TAC.

## Abstract

**Background:**

Recent studies have shown that oxidative stress (OS) is the most important indicator in the pathogenesis of Alzheimer's disease (AD), but the results in previous studies are conflicting. This study aimed to assess the plasma levels of 8-hydroxy-2'-deoxyguanosine (8-OHdG) as DNA oxidative damage marker and total antioxidant capacity (TAC) in patients with AD versus control group.

**Methods:**

Thirty patients with AD and 30 sex-and age-matched healthy subjects were studied. Diagnosis of AD was based on National Institute of Neurological and Communicative Disorders and Stroke and the Alzheimer's disease and Related Disorders Association (NINCDS/ADRDA) criteria. Also for the patients, Mini-Mental State Examination (MMSE), computed tomography (CT) scan and brain magnetic resonance imaging (MRI) were done. Plasma levels of 8-OHdG and TAC were measured by competitive ELISA method and ferric reducing antioxidant power (FRAP) assay, respectively.

**Results:**

Plasma levels of 8-OHdG was significantly higher in AD compared to control group (p<0.001), while the total antioxidant was significantly lower in patients compared to controls (p=0.002). The value of area under the ROC curve for 8-OHdG and TAC in discriminating AD from controls were 0.87 and 0.32, respectively.

**Conclusion:**

Our results indicate a link between oxidative stress and AD indicating a possible contributive role of these markers in the development of AD and as an indicator in the discrimination of AD from healthy controls.

Alzheimer's disease (AD) is an advanced dementia that affects a large number of aging people (1) and characterized by loss of memory, decrease of function and behavioral disorders which affects a significant proportion of elderly subjects (1, 2). Neuronal cell death, formation of amyloid plaques, and neurofibrillary tangles are histopathological alterations in AD (1). According to recent studies, more than 18 million people have been affected with AD worldwide and this number will almost double by 2050 (3). Evidence demonstrated that aging is a major risk factor of AD which is associated with increased reactive oxygen species (ROS) (4). Oxidative stress, a major and one of the most important factors in the pathogenesis of AD (5). Stress oxidative occurs when the production of reactive oxygen species (ROS) and reactive nitrogen species (RNS) increases and/or antioxidant defense system decreases (6, 7). Our previous studies demonstrated that ROS damages macromolecules such as lipids and proteins in several regions of the rat brain and causes changes in their structure and function (8-10).

8-OHdG is the most common marker of DNA oxidation produced by oxidation of DNA bases (5). Considering the importance of oxidative stress in Alzheimer's disease, therefore, we aimed to determine the plasma levels of 8-OHdG and TAC in AD patients and healthy controls. These data may provide additional data in the elucidation of pathogenesis as well as prevention of AD. In addition, reduction of oxidative stress may be helpful in the treatments for AD.

## Methods

 This case-control study was done on 30 patients with severe AD and 30 healthy subjects as the control group from Mehr-Avaran Shomal Nursing Home, Sari, Iran. The control and patient groups were matched by sex and age. Alzheimer's patient diagnosis was based on the National Institute of Neurological and Communicative Disorders and Stroke and the Alzheimer's disease and Related Disorders Association (NINCDS/ADRDA) criteria and the Mini-Mental State Examination (MMSE) score of 0-10, computed tomography (CT) scan and brain magnetic resonance imaging (MRI) by a neurologist. Other causes of dementia were ruled out by imaging and laboratory tests. The control group had normal MMSE scores.

 The study protocol was approved by the Ethics Committee of the Babol University of Medical Sciences. Informed consent was obtained from the comparisons or relatives of all patient participants. Subjects with inflammatory and infectious diseases such as anemia, diabetes, hepatitis, and also consumers of antioxidant and vitamins, and folic acid supplementations were excluded from our study. The blood samples were collected in sodium heparin tubes from fasting subjects. The separation of plasma was done by centrifugation at 1500 rpm for 10 min and stored at -80°C until measurement.

**Measurement of Total Antioxidant Capacity (TAC): **The ferric reducing antioxidant power (FRAP assay) is a method to determine TAC (total antioxidant capacity). According to this method, a blue ferrous complex is formed by reduction of colorful ferric-tripyridyltriazine complex in the presence of antioxidant. The absorbance of samples was determined at a wavelength of 593 nm. After comparing the absorbance of each sample to the standard curve, the quantity of antioxidant power was calculated and the TAC has reported in μmol/L (11).

**Measurement of 8-hydroxy-2'-deoxyguanosine (8-OHdG): **The 8-OHdG concentration was analyzed by competitive ELISA kit (Chongqing Biospes CO., Ltd). The unknown 8-OHdG samples or 8-OHdG standards were first added to an 8-OHdG/BSA conjugate preabsorbed ELISA plate. After a brief incubation, an anti-8OHdG monoclonal antibody was added, followed by an HRP conjugated secondary antibody. 

After the addition of substrate solution to each well, a blue color was produced. Then the ELISA plate was incubated at room temperature for 30 min. Finally, stop solution was added and a yellow color was produced. The absorbance of samples were measured at 450 nm. The 8-OHdG content in unknown samples was determined by comparison with predetermined 8-OHdG standard curve. The concentration of 8-OHdG was reported in ng/ml (12).

**Statistical analysis: **Statistical analysis were done using SPSS Version 18. Results were expressed as mean±SEM for all parameters. The data analyses were performed using independent t-test. Also, Mann- Whitney test was used for data analysis with abnormal distribution. The receiver operative characteristic (ROC) curve was used for discrimination of AD patients from healthy controls. A p-value<0.05 was considered statistically significant.

## Results

Demographic and medical features of the patients and control groups were shown in table 1. The two groups were similar in regard to age and sex (p>0.05) but significantly different regarding MMSE (p<0.001). The mean value of plasma 8-OHdG levels in AD was significantly higher than the control group (P<0.001). 

**Figure1:**
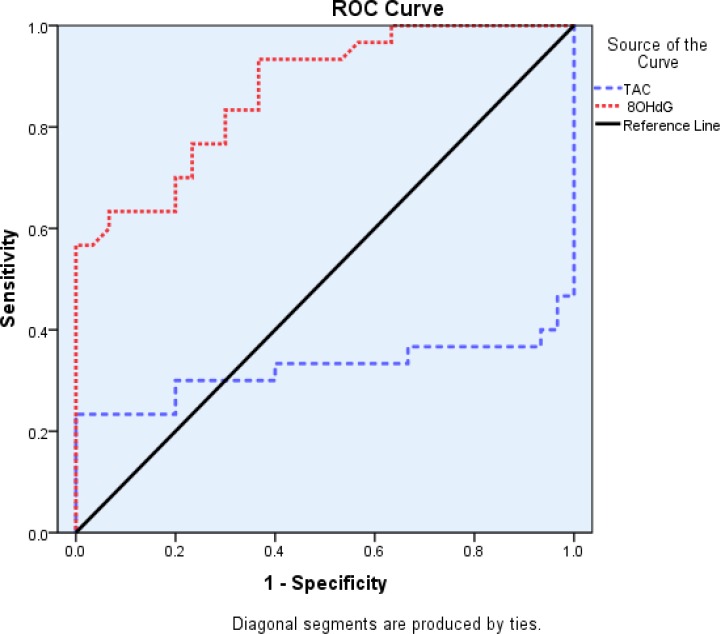
The area under ROC curve for 8-OHdG and TAC in plasma of Alzheimer patients

While the TAC levels (P=0.002) in patients were significantly lower than controls (Table 2). The area under ROC curve (AUC) in plasma of patients for 8-OHdG and TAC were (0.87 and 0.32, respectively) (figure 1 and table 3).

**Table 1:** Demographic and medical features of the patients and control groups

** Group** **Variable**	**Patient(n=30)** **Mean±SEM**	**Control(n=30)** **Mean±SEM**	**P value**
Age (years)	81±1.51	77.8±1.10	0.09
Sex (F/M)	22.8	22.8	1.000
MMSE	3.6±0.24	28.3±0.77	<0.001

Abbreviations: MMSE, Mini-Mental State Examination

**Table 2:** Mean±SEM of oxidative stress markers in plasma of Alzheimer patients and control groups

**Markers**	**Mean±SEM**	**P value**
**8-OHdG (ng/mL) **		
Case (n=30) Control (n=30)	957.03±52.41 572.23±35.06	<0.001
**TAC (μmol/L)**		
Case (n=30) Control (n=30)	1140.63±69.03 1364.48±12.62	0.002

Abbreviations: 8-OHdG, 8-hydroxy-2'-deoxyguanosine; TAC, Total antioxidant total; MMSE, Mini-Mental State Examination.

**Table 3:** Area under the ROC curve (AUC) of oxidative stress markers and total antioxidant capacity in plasma of Alzheimer patients and control subjects

**Asymptotic 95% CI**		**Asymptotic sig. **	**Std. Error**	**AUC**	**Markers**
**Upper bound**	**Lower bound**				
0.957	0.785	0.000 **	0.044	0.87	8-OHdG
0.479	0.165	0.018 *	0.080	0.32	TAC

* P< 0.05,

** P< 0.0001

## Discussion

Our finding demonstrated a significantly higher plasma 8-OHdG concentration but significantly lower total antioxidant in AD versus healthy controls. Several studies reported similar results. In a study, Mecocci P. et al. evaluated the levels of 8-OHdG on lymphocytes in AD patients and controls. They reported a significantly high 8-OHdG levels in the patients than controls (4). Similar to a previous study, Mecocci P et al. determined the status of lymphocytes 8-OHdG levels in AD patients and controls and reported that the levels of 8-OHdG were significantly higher in the patients (13).

In one study on 13 healthy and 13 AD patients, the level of 8-OHdG marker was assessed in the three regions of cerebral cortex and cerebellum in patient’s brain and the results showed a statistically significantly threefold increase in mitochondrial DNA (mtDNA) 8-OHdG levels in patients in comparison with the controls (14). This marker was also detectable in peripheral cells (13). 

Since some antioxidants protect DNA from oxidative damage. Therefore, the increased 8-OHdG levels may be due to decrease in plasma antioxidants (4). Results of ROC analyses revealed that 8-OHdG can be considered as a possible marker to distinguish AD from healthy people. On the other hand, in the present study, total antioxidant capacity (TAC) levels significantly decreased in patients with AD in comparison to controls and this marker was evaluated by FRAP. 

Critalli et al. assessed the plasma level of TAC in AD patients (mild, intermediate and advanced disease) versus control groups. They found significantly lower TAC in AD patients (all stages) as compared to control group (15). Also, Aldred et al. showed a significant changes in TAC (decrease) in severe AD with respect to controls (16). In another study, a significant decrease of TAC levels in AD patients was reported (17). Zifrilla et al. observed low levels of plasma TAC in light-moderate and severe AD groups when compared to healthy control (18).This reduction might be due to malnutrition and high-speed production of free radicals in patients (4). 

In contrast to the reported studies, some studies have not found a significant difference in plasma TAC between the AD and control groups (19, 20). According to the results obtained from our study, it seems the AD in advanced stages have higher levels of oxidative stress and lower levels of antioxidants. We demonstrated weak antioxidant defense system in patients with severe AD. Also, the level of DNA oxidative damage marker was higher. These subjects were very old with severe stage. So, lower levels of antioxidants and increased oxidative stress index may also be attributed to old age, malnutrition, lifestyle and irregularities in the antioxidant defense system. Since the subjects of the control group were at similar in age, thus the confounding effect of age on the results of this study should be ignored. However, although the patients with coexistent apparent clinical diseases were excluded, the influences of other factors particularly asymptomatic coexistent common chronic medical conditions such as diabetes, cardiovascular, chronic muscoloskeletal disorders can not be ignored. 

In Conclusion, the results of this study indicate a link between the damage caused by oxidative stress and AD and 8-OHdG marker may be used to differentiating subjects with and without AD. These findings need to be confirmed by further prospective longitudinal studies with adequate sample size.

## Notes

**Funding:** This work was financially supported by Babol University of Medical Sciences.
